# Failures

**Published:** 1870-04

**Authors:** 


### FAILURES.

We recently heard a professional brother exceedingly anxious to tell all his failures in practice. At the time it just occurred to us, that if he should really do what he seemed so anxious for, he would have his hands full.

We frequently hear in our Associations, members of the profession, detailing their failures in practice, and apparently very anxious that everybody else should do the same thing. We confess, that we are not quite yet in the proper state of illumination, or befogment, to understand the propriety of becoming ecstatic over our failures.

We have usually found that it is an easy matter to make a failure, almost anybody can do that; and, besides, there are a great many ways of making failures, and but few of securing success; indeed, in some things, there is but one way of being successful, while there may be a hundred of making failures. Our seeking and investigation should always be for the right way; if one starts out upon a journey, it would hardly be accounted wise for him to enquire for all the wrong roads, those of a wrong direction, the rough, thorny, stony, boggy, dark and dangerous ways; but it would be the part of wisdom to get in the right way, the bright way, and the successful way, and keep in it. But, says our failure man, "I'm on the wrong road, I want to be put right; I'm in the dark, I want light." Well, brother, you should not get out of the right way, you should not get in the dark; the way is plain—the glorious noonday light beams all around us, and if we will but open our eyes, and remove the scales of ignorance, we shall see clearly and distinctly all about us, and we will find the way so clearly indicated, that "he who runs may read."

Let us talk of our successes, and tell how we accomplish them, and cast our failures with the darkness and ignorance that attaches to them—to the moles and bats. T.

